# Antimicrobial Efficacy of a Novel Automated Irrigation Device As Compared to Conventional Needle Irrigation Against Enterococcus faecalis: An In Vitro Study

**DOI:** 10.7759/cureus.45200

**Published:** 2023-09-14

**Authors:** Sahil Choudhari, Pradeep S, Kavalipurapu Venkata Teja

**Affiliations:** 1 Department of Conservative Dentistry and Endodontics, Saveetha Dental College, Saveetha Institute of Medical and Technical Sciences (SIMATS) Saveetha University, Chennai, IND

**Keywords:** root canal, manual irrigation, irrigants, enterococcus faecalis, automated irrigation

## Abstract

Aim

The present study aims to compare the antibacterial efficacy of a novel automated endodontic irrigation device with that of a manual irrigation technique.

Materials and methods

The present study considered 45 extracted single-rooted teeth. After sectioning the teeth, the specimens were inoculated with *Enterococcus faecalis* (*E. faecalis*) bacteria for three weeks. Instrumentation was done using ProTaper Gold rotary files to size 50 and 5% taper. Based on the irrigation protocol, the experimental samples were divided into Group I: control (normal saline irrigation) (n = 15); Group II: conventional needle irrigation (n = 15); and Group III: automated irrigation (n = 15). The irrigation in Group III was done using the automated irrigation device. After the experimental irrigation, the pre-weighted dentinal shaving was collected in Eppendorf tubes, diluted 10 times, and incubated in the Petri dish with tryptone soy agar (TSA) for 48 hours. Finally, the colony-forming unit (CFU) counts were assessed. IBM SPSS Statistics for Windows version 23.0 (Armonk, NY, USA, IBM Corp.) was used for data analysis. Intergroup comparisons were made using the non-parametric Kruskal-Wallis test.

Results

The mean CFU count (CFU/ml) for the groups was: Group 1 (normal saline irrigation): 3.67x10^1^; Group 2 (manual irrigation): 2.69× 10^1^; Group 3 (automated irrigation): 1.57× 10^1^. A statistically significant reduction in *E. faecalis* CFU count was seen among the groups assessed (p<0.01). The automated irrigation group exhibited the most substantial reduction in *E. faecalis* CFU count. Group 2 showed a significant difference compared to Group 1 (p<0.05).

Conclusions

The novel automated endodontic irrigation device was superior to manual irrigation in reducing *E. faecalis* CFU counts. This indicates that the automated irrigation device has the potential to enhance bacterial elimination efficacy during endodontic treatment.

## Introduction

Endodontic therapy should aim at the successful elimination of bacteria and their contents from the canal [1]. Around 40%-60% of cultivable bacteria are seen even after sodium hypochlorite irrigation [2,3]. Biofilm habituation in the root canal is the primary cause of pathogenesis. Incomplete removal of these microorganisms during endodontic treatment can result in infection, which tends to be majorly responsible for root canal treatment failures [4]. *Enterococcus faecalis* (*E. faecalis*), a gram-positive facultative anaerobic bacterium, is often encountered in retreatment cases [5]. Therefore, the current study aimed to target *E. faecalis* as a test organism to assess the antibacterial efficiency of different irrigation regimens. Moreover, the root canal system's dentin composition and anatomical complexities in persistent infection tend to limit root canal disinfection [6].

Although previous literature focused on additional intracanal medication after debridement of infected teeth [7], current systematic review evidence claims no real benefit of using the intracanal medication in teeth with apical periodontitis [8]. Hence, there is a need for newer or alternative root canal irrigation techniques for effective microbial reduction. Studies also claim the development of resistant species by using intracanal medication for infected cases [9]. Hence, there is an urge to assess the newer root canal irrigation techniques.

The syringe needle irrigation (SNI) technique is the most commonly employed technique for root canal irrigation. It is claimed to be the most commonly employed irrigation technique by clinicians [10]. However, it is claimed to have its own disadvantages, which make its clinical use cumbersome [11,12]. Hence, the recently introduced automated root canal irrigation device by Teja et al. (Indian Patent Copyright Application Number 201941037185) could be a potent alternative to the currently used manual irrigation techniques [13]. Current literature also claims the complexity of root canal irrigation and notes various parameters enhancing the efficacy of syringe needle irrigation [12]. Hence, the operating clinician cannot maintain standard flow rates and pressures during syringe needle irrigation [13]. Hence, the automated irrigation device could deliver the irrigation fluid at a constant flow rate, thereby enhancing the irrigation efficacy. However, there is no clear scientific evidence for the use of this device for clinical purposes.

When* E. faecalis* reduction is considered, various irrigants [14], irrigation techniques [15], and the usage of activation devices [16] have been explored in depth. However, there are no reports on using alternative syringe needle techniques. Hence, in the current study, we aimed to assess the real benefit of employing the automated root canal device as an alternative to manual irrigation techniques. When the specific bacterial species is considered, *E. faecalis* is considered to be a potent bacterial species isolated even from failed root canals [17]. Hence, the current study aimed at assessing the efficacy of an automated irrigation device on *E. faecalis* reduction. The tested null hypothesis was that there was no significant difference in the E. faecalis reduction when using automated irrigation devices as compared to SNI.

## Materials and methods

An in vitro study was planned following the IRB ethical approval (IHEC/SDC/ENDO-1713/21/168) from the institutional human ethical committee of Saveetha Dental College and Hospitals, Saveetha Institute of Medical and Technical Sciences, Chennai, India. The sample size for the present study was obtained based on Ivana Tolijan et al. [18]. A total sample size of 45 (1-β = 90%, α = 0.05, f = 0.55) was obtained based on the calculation.

In the current study, 45 single-rooted extracted mandibular premolars with mature apices were selected. Teeth with root caries, curved canals, and canal calcifications were excluded. The single canal morphology was confirmed with intraoral periapical radiographs taken at different angulations. Access preparation was performed with a high-speed Endo-Access bur (Dentsply Maillefer Instruments, Ballaigues, Switzerland), and walls were refined by an Endo-Z bur (Dentsply Maillefer Instruments, Ballaigues, Switzerland). Patency of the root canal was achieved using the ISO No. 10 hand K file. Instrumentation of the root canal was done to a size of 50 and 5% taper at the working length using ProTaper Gold rotary files (Dentsply Maillefer Instruments, Ballaigues, Switzerland). Following shaping, a rotary diamond disc was used to decoronate the specimen to obtain 6 mm of the middle third of the root.

The external diameter was standardized to 4 mm by removing the cementum from the root surface [19]. Then, using Gates Glidden drill no. 3 (Mani Inc., Tochigi-ken, Japan), the internal diameter was standardized using a slow-speed handpiece (Nippon Seikō Kabushiki-gaisha (NSK), Tokyo, Japan); 3% sodium hypochlorite (NaOCl) (Prime Dental Product, Mumbai, India), followed by 17% ethylenediamine tetraacetic acid (EDTA) (Prime Dental Product, Mumbai, India), were used for five minutes each to remove the organic and inorganic debris from the specimens. Next, the teeth were placed for 10 minutes in distilled water to remove the traces of chemical remnants and then subjected to an autoclave at 121°C for 20 minutes [20].

After instrumentation of the root canal, the specimens were contaminated with an *E. faecalis* (ATCC 29212) strain, which was used as a test organism in the current study. It was grown on tryptone soya agar (TSA), suspended in 5 ml of tryptic soy broth (TSB), and incubated for four hours at 37°C. The turbidity was equivalent to 0.5 McFarland standards. 50 μL of the *E. faecalis* inoculums were transferred into pre-sterilized microcentrifuge tubes containing 1 ml of tryptone soy broth. The dentin specimens were transferred into the fresh broth containing *E. faecalis* at the end of 24 hours and maintained under laminar flow (Clean Air, Mumbai, India). The culture purity was confirmed by subculturing 5 μL of the broth from the incubated dentin specimens in TSB on tryptone soy agar plates. The samples were incubated with *E. faecalis* at 37°C for 21 days [21]. The entire access cavity preparation, root canal shaping, and specimen contamination procedures were performed by an endodontist (S.S.), who was not involved in the study.

After 21 days of the incubation period, the specimens were rinsed with 5 ml of sterile water to remove the inoculated broth and were assigned randomly by a head nurse using the computer-generated randomization technique (www.random.org) into three groups based on the irrigation protocol:

Group 1: control (distilled water irrigation) (n = 15) Group 2: conventional needle irrigation (n = 15) Group 3: automated irrigation (n = 15)

The entire irrigation protocol was performed by a single operator, who was blinded until the end of the study. Once the operator was provided with the respective specimens, instructions on the protocols were provided. Based on the respective groups, the protocols differed.

Group I: control (normal saline irrigation)

The samples were irrigated using 20 ml of normal saline with a 30-gauge side-vented needle. (NaviTip, Ultradent Products, South Jordan, USA), placed 1mm short of the working length.

Group II: conventional needle irrigation

Irrigation was carried out using 20 ml of 5% NaOCL. The final rinse was carried out using 5 ml of saline. The entire irrigation in this group was carried out using a 30-gauge side-vented needle attached to a 5-ml syringe barrel.

Group III: automated irrigation

**Figure 1:**
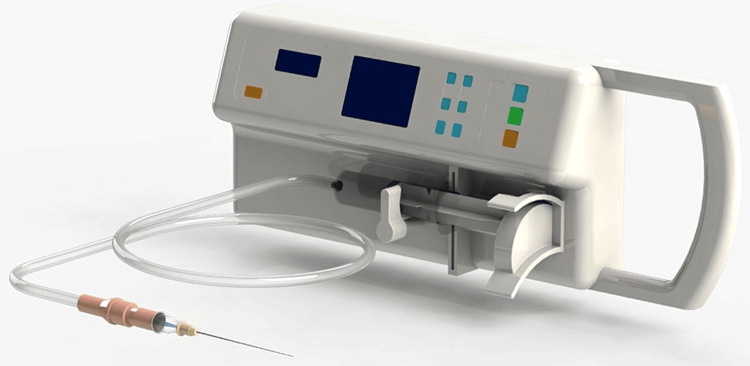
A novel automated endodontic irrigation device prototype

The irrigation in this group was carried out using the patented automated irrigation device. The primary working mechanism of this device is based on rate delivery mode. The irrigant is delivered at a specified flow rate. The rate modes possible in the current device ranged from 0.5 to 12 ml/min. The irrigation protocol was performed by attaching a 30-gauge side-vented needle to a disposable 5-ml plastic syringe, and the entire customized set was coupled to the automated root canal irrigation device (Figure 1).

The irrigation was carried out using an automated root canal device at a flow rate of 6 ml/min. Separate 5-ml syringe barrels and irrigation needles were used for the respective irrigants to prevent contamination and inherent precipitation formation. A total of 20 ml of 5% NaOCL irrigation was carried out using an automated irrigation device. The final rinse was carried out using 5 ml of saline.

**Figure 2:**
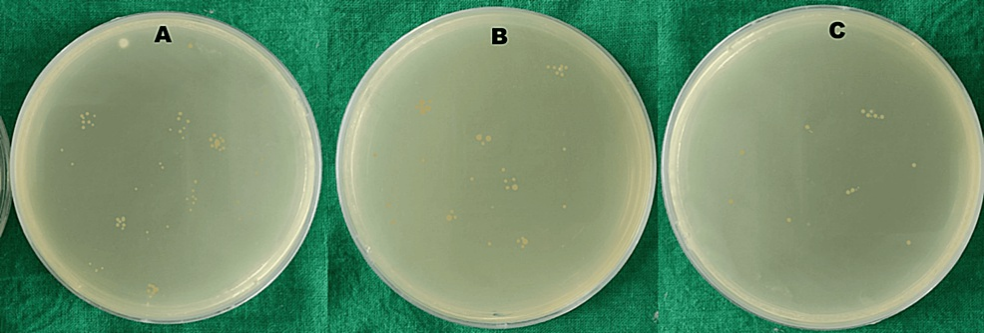
Colony-forming unit counts of the various tested groups: A: Group I (control), B: Group II (manual irrigation), and C: Group III (automated irrigation).

Once the entire irrigation was completed, the dentin shavings obtained from the inner third of the dentin were obtained using Gates Glidden (GG) 1 to the apical depth, followed by GG 2, 3, 4, and 5. The pre-weighed shavings were then immersed in 1 ml of TSA broth in 1.5 ml Eppendorf tubes (Eppendorf, Hamburg, Germany). The tubes were vibrated on Fisher Vortex equipment (Thermo Fisher Scientific Inc., Waltham, USA) for two minutes to homogenize the samples. Ten-fold dilutions were prepared, and 1 ml of aliquots of suspensions were seeded on a Petri dish with TSA plates and then incubated at 37°C for 48 hours. The colony-forming units (CFU) grown were counted using a stereomicroscope, and log transformation was carried out for the values obtained (Figure 2, Table 1).

**Table 1:** Table depicting the mean reduction of CFU count (Enterococcus faecalis) in different groups Table 1 infers the mean reduction of CFU count; Group 3 showed a statistically significant difference compared to Group 2 and Group 1 (< 0.01). Group 2 showed a significant difference compared to Group 1 (<0.01). CFU: colony-forming unit

Groups	Mean (x10^1^)	Std. Deviation (x10^1^)	p Value
Group 1	3.67	0.39	<0.01
Group 2	2.69	0.16	
Group 3	1.57	0.59	

Statistical analysis

IBM SPSS Statistics for Windows version 23.0 (Armonk, NY, USA, IBM Corp.) was used for data analysis. The non-parametric Kruskal-Wallis test was used for the statistical analysis. The data was tabulated in a Microsoft Excel sheet (Microsoft Corporation, WA, Redmond, USA).

## Results

In Group 3, irrigation was conducted using a new automated irrigation device, delivering a flow rate of 6 ml/min (Figure 1). When evaluating the mean CFUs across the three groups, the results were as follows: Group 1 had a mean CFU of 3.67x 101 CFU/ml, Group 2 had a mean CFU of 2.69× 101 CFU/ml, and Group 3 exhibited the lowest mean CFU at 1.57× 101 CFU/ml.

There was a substantial reduction in bacterial counts, and this reduction was statistically significant (p<0.01) when comparing the different groups, as illustrated in Figure 2 and detailed in Table 1. Notably, Group 3 demonstrated the most substantial reduction (P<0.01) in *E. faecalis* CFU count compared to the other groups. Furthermore, Group 2 also exhibited a significant difference compared to Group 1 (p<0.01). These findings highlight the effectiveness of the novel automated irrigation device in significantly reducing* E. faecalis* bacterial counts during the study.

## Discussion

The present study is intended to assess different irrigation strategies for reducing the *E. faecalis* load in infected specimens. The results showed a significant difference (p<0.05) in the automated irrigation device as compared to SNI in reducing the bacterial load. So, the currently proposed device could be an alternative to the currently used manual syringe needle irrigation technique.

Previous data show the importance of increasing the master apical file size in improving healing outcomes in cases with apical periodontitis [22]. The mechanism understood from such a theory was that more dentin removal led to more clean shavings from the apical third, leading to better disinfection. However, the current concepts are clear in terms of minimal debridement and maximal disinfection strategies [23].

When debridement strategies for bacterial removal using newer irrigation devices have to be assessed, current literature favors the usage of passive ultrasonic irrigation as compared to the other irrigation regimens to date [21,24]. However, to date, there are no alternatives available for manual irrigation techniques, which are usually performed clinically using a syringe and barrel. There are also literature reports that prove the inefficiency of SNI alone in *E. faecalis* reduction [21]. So, taking all these factors into consideration, the present study focused on assessing the efficacy of an automated irrigation device on *E. faecalis* reduction.

All the parameters in the present study were standardized. The entire procedure was performed by a single blind operator. The entire protocol was similar in both experimental groups, except for the use of different devices for irrigation. Normal saline irrigation was used as a control in the present study to compare the real benefits of using the proposed experimental regimen. Although the minimum and maximum possible flow rates of 1 ml/min and 15 ml/min are possible with SNI, the flow rate during automated irrigation was set at 6 ml/min. The reason for choosing such a flow rate was based on a previous ex-vivo study that proved the physiological apical pressure limit to be 1-6 ml/min [21,25]. Hence, we considered such a flow rate for our study. As far as the study results are concerned, as the irrigant volume and the regimen were standardized in both groups, there might not be a notable variation in the significance observed in both experimental groups.

When the limitations of the present study are considered, colony-forming units assessing *E. faecalis* might not indeed translate into a clinical condition. Ideally, endodontic infections are polymicrobial and caused by multispecies biofilm. Hence, future studies are advised to be carried out by considering all these factors.

## Conclusions

Overall, the outcomes of this study highlight the potential of the automated irrigation device in improving endodontic bacterial elimination efficacy. The novel endodontic irrigation device has proven to be more efficient than manual irrigation in reducing *E. faecalis* CFU counts. Successful outcomes in endodontic treatments are essential, and this automated irrigation device has the potential to improve treatment efficacy and reduce failures. Future studies should focus on assessing the device's efficacy with various activation systems to prove its clinical applicability.
